# Heteronanostructured Co@carbon nanotubes-graphene ternary hybrids: synthesis, electromagnetic and excellent microwave absorption properties

**DOI:** 10.1038/srep37972

**Published:** 2016-11-28

**Authors:** Xiaosi Qi, Qi Hu, Hongbo Cai, Ren Xie, Zhongchen Bai, Yang Jiang, Shuijie Qin, Wei Zhong, Youwei Du

**Affiliations:** 1 College of Physics, Guizhou University, Guiyang 550025, People’s Republic of China; 2 Collaborative Innovation Center of Advanced Microstructures, Nanjing National Laboratory of Microstructures and Jiangsu Provincial Laboratory for NanoTechnology, Nanjing University, Nanjing 210093, People’s Republic of China

## Abstract

In order to explore high efficiency microwave absorption materials, heteronanostructured Co@carbon nanotubes-graphene (Co@CNTs-G) ternary hybrids were designed and produced through catalytic decomposition of acetylene at the designed temperature (400, 450, 500 and 550 °C) over Co_3_O_4_/reduced graphene oxide (Co_3_O_4_/RGO). By regulating the reaction temperatures, different CNT contents of Co@CNTs-G ternary hybrids could be synthesized. The investigations indicated that the as-prepared heteronanostructured Co@CNTs-G ternary hybrids exhibited excellent microwave absorption properties, and their electromagnetic and microwave absorption properties could be tuned by the CNT content. The minimum reflection loss (RL) value reached approximately −65.6, −58.1, −41.1 and −47.5 dB for the ternary hybrids synthesized at 400, 450, 500 and 550 °C, respectively. And RL values below −20 dB (99% of electromagnetic wave attenuation) could be obtained over the as-prepared Co@CNTs-G ternary hybrids in the large frequency range. Moreover, based on the obtained results, the possible enhanced microwave absorption mechanisms were discussed in details. Therefore, a simple approach was proposed to explore the high performance microwave absorbing materials as well as to expand the application field of graphene-based materials.

Microwave absorbing materials (MAMs) have attracted more and more attention over the world due to the expanded electromagnetic (EM) interference problems to electronic communication devices and invisible harm to biological system^1^,^2^,^3^,^4^. According to EM energy conversion principle, the reflection coefficient of an ideal absorber should be zero when it fulfills the optimal EM impedance matching condition^5^,^6^,^7^. However, single component dielectric loss material such as ZnO or magnetic loss material such as Fe_3_O_4_ is very difficult to meet this condition due to the mismatch in the values of complex permittivity 
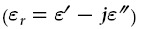
 and complex permeability 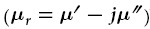
8,9,10. Based on the impedance matching strategy, one of the effective ways to solve the problem is to couple dielectric materials with magnetic nanoparticles. Among these hybrids, heteronanostructured hybrids have been proved to exhibit strong EM wave absorption properties because of their interfacial and synergistic effects. Therefore, different categories of heteronanostructured hybrids were developed to further improve their EM wave absorbing capabilities, such as Fe@ZnO^11^, Fe_3_O_4_@SiO_2_^12^, Fe_3_O_4_@SnO_2_^13^, FeCo@ZnO^14^, Ni@TiO_2_^15^, and so on^16^,^17^,^18^,^19^. Generally, the previously reported results indicated that the microwave absorption (MA) capability of MAMs was mainly determined by the ***ε***_***r***_, ***μ***_***r***_, EM impedance matching and microstructure. However, these hybrids still suffer from some problems such as high density, large thickness of absorber, optimal RL usually above −40 dB, and so on. Currently, high performance MAMs with light weight, thin thickness, good chemical stability, strong absorption ability and wide absorption frequency are highly desired.

Graphene-based materials (graphene, graphene oxide, etc), have become the current focus because of their outstanding physical and chemical properties such as the excellent thermal and electronic conductivity, huge specific surface area, and strong mechanical strength^20^,^21^,^22^,^23^. The unique and fascinating properties make graphene and graphene-based hybrids become the promising candidates in the fields of gas sensing^24^, field-effect transistors^25^, supercapacitors^26^, and MAMs^27^. Unfortunately, graphene-based materials alone cannot achieve ideal MA properties because of the mismatch between relatively high dielectric loss and low magnetic loss^28^,^29^. The incorporation of magnetic materials into graphene-based materials is an effective strategy to obtain excellent MA properties, and previous theoretical studies indicate that the interfacial electronic interaction between metal and graphene can make graphene show some novel magnetic and electric properties^30^,^31^. Therefore, different categories of magnetic particles-graphene based hybrids have been developed to improve MA properties in the recent years^32^,^33^,^34^,^35^. Herein, we design and synthesize a new Co@carbon nanotubes-graphene (Co@CNTs-G) ternary hybrid. The aim of designing these hybrids has mainly three points: firstly, Co nanoparticles and CNTs/graphene can be used as magnetic and dielectric loss materials, respectively. By controlling their compositions, a good impedance matching may be obtained on these hybrids. Secondly, both the tube and layer structures of CNTs and graphene are very beneficial to improve MA performance of hybrids in theory. Finally, the heteronanostructure of hybrid can provide the synergetic effect of Co, CNTs and graphene, which should enhance MA capability greatly.

## Results

**Figure 1:**
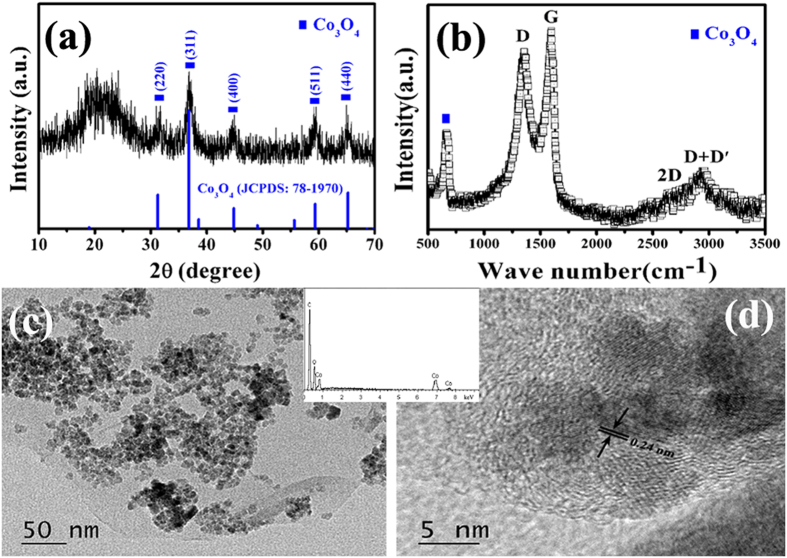
(**a**) XRD pattern, (**b**) Raman spectrum, (**c**) TEM image, and (**d**) HRTEM image of Co_3_O_4_/RGO (inset: EDS result).

Figure 1 presents the XRD pattern, Raman spectrum and TEM images of Co_3_O_4_/reduced graphene oxide (Co_3_O_4_/RGO). As shown in Fig. 1a, the diffraction peaks (as indicated by the symbols) at 31.3, 36.8, 44.8, 59.3 and 65.2° can be assigned to (220), (311), (400), (511) and (440) crystal planes of the face-centered cubic phase Co_3_O_4_ (JCPDS: 78–1970), respectively. And the broad diffraction peak at ca. 21.9° is attributed to the graphite-like structure, which should be indexed to RGO^36^,^37^. In order to obtain more information about RGO, the Raman spectrum of Co_3_O_4_/RGO is presented in Fig. 1b, two strong peaks centered at ca. 1348 (D band) and 1598 cm^−1^ (G band) can be observed clearly. As we know that the D band is related to the defect or disorder in the graphitic structure and G band is indicative of high crystallinity graphitic layer^38^,^39^,^40^. The peaks appeared in the range of 2500–3000 cm^−1^ are characteristic Raman signal of RGO^41^,^42^. Additionally, the A_1g_ peak of Co_3_O_4_ can also be seen at ca. 670 cm^−1^. The morphology of Co_3_O_4_/RGO was investigated by TEM. As shown in Fig. 1c, large quantities of Co_3_O_4_ nanoparticles are anchored on RGO surface without serious aggregation. The high-resolution TEM (HRTEM) image (as shown in Fig. 1d) exhibits a clear crystal lattice with a interplanar spacing of 0.24 nm, which corresponds to the (311) plane of Co_3_O_4_. The energy dispersive X-ray spectroscopy (EDS) result (as shown in the inset of Fig. 1) indicates that the catalyst precursor (Co_3_O_4_/RGO) contains C, Co and O, and their atomic percentage is ca.69:3:28, which implies that RGO should contain a certain amount of oxygen. Generally, all the obtained results indicate that the catalyst precursor is Co_3_O_4_/RGO.

**Figure 2:**
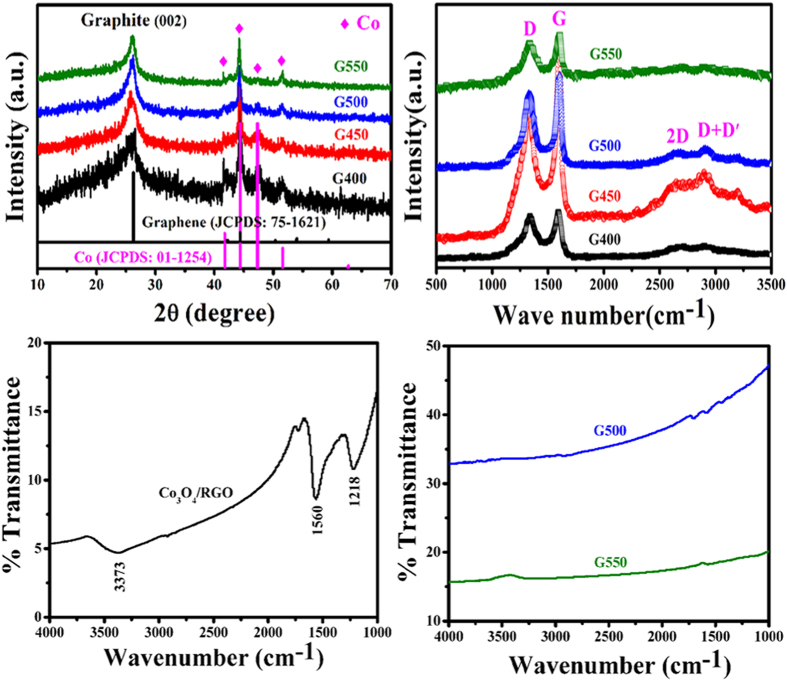
(**a**) XRD patterns, (**b**) Raman spectra, and (**c,d**) FT-IR spectra of Co_3_O_4_/RGO and the as-prepared hybrids.

Based on our previous works^43^,^44^,^45^, the method of chemical vapor deposition (CVD) was used to synthesize Co@CNTs-G ternary hybrids via catalytic decomposition of acetylene at the selected temperature over Co_3_O_4_/RGO. As depicted in experimental section, compared to the amount of Co_3_O_4_/RGO, much higher quantities of samples could be collected after the designed CVD processes. In order to investigate their phases and microstructures, the obtained samples were characterized by XRD, Raman, FT-IR, SEM and TEM. Figure 2 shows the XRD patterns, Raman and FT-IR spectra of the obtained Co_3_O_4_/RGO and Co@CNTs-G. As shown in Fig. 2a, the diffraction peaks of the as-prepared Co@CNTs-G ternary hybrids (G400, G450, G500 and G550) located at ca. 41.8, 44.4, 47.3 and 51.6° can be assigned to the phase of Co (JCPDS: 01–1254). And the diffraction peak centered at ca. 26.2° can be assigned to the (002) crystal plane of hexagonal phase graphite (JCPDS: 75–1621). The comparison XRD results (as shown in Figs 1 and 2) indicate that the sample undergoes a transform process from RGO and Co_3_O_4_ to graphene and Co, respectively, through the annealing treatment of Co_3_O_4_/RGO in acetylene. Moreover, one can find that the relative XRD peak intensity ratio of Co and graphene is decreasing with the increased temperature, which should be related to the different Co contents of the as-prepared Co@CNTs-G ternary hybrids. In our experiment, the obtained experimental results indicated that over the same amount of Co_3_O_4_/RGO (0.02 g), much larger quantities of Co@CNTs-G could be collected at the higher temperature, which is consistent with the different intensity ratios of Co and graphene in XRD patterns. Figure 2b presents the Raman spectra of the obtained Co@CNTs-G ternary hybrids. Similar to that of Co_3_O_4_/RGO (as indicated in Fig. 2b), four peaks can be observed clearly, which are indexed to D, G, 2D (intrinsic peak of graphene) and D + D′ bands, respectively. Combined with the XRD results, the existence of 2D and D + D′ bands indicates the presence of graphene. Comparison of the obtained Raman results, it can be seen that the 2D and D + D′ bands almost cannot be observed over the obtained G550, which may be related to the low content of graphene in the obtained hybrid. In order to detect the degree of removing the oxygen-containing functional group^46^,^47^,^48^, the FT-IR spectra of Co_3_O_4_/RGO, G500 and G550 are shown in Fig. 2c and d. As shown in Fig. 2c, the broad absorption band observed at 3373 cm^−1^ corresponds to the stretching vibration of intermolecular hydrogen bond O-H, and the characteristic peaks appear at 1560 and 1218 cm^−1^ are due to the stretching vibration of C=C and epoxy C-O, respectively. The detection of oxygen-containing functional group over Co_3_O_4_/RGO is consistent with the EDS result (as shown in Fig. 1). And no IR signals such as –OH and C-O are detected over the as-prepared Co@CNTs-G (as shown in Fig. 2d). These results point out that the chemical reduction of Co_3_O_4_/RGO with C_2_H_2_ significantly reduces the oxygen species.

**Figure 3:**
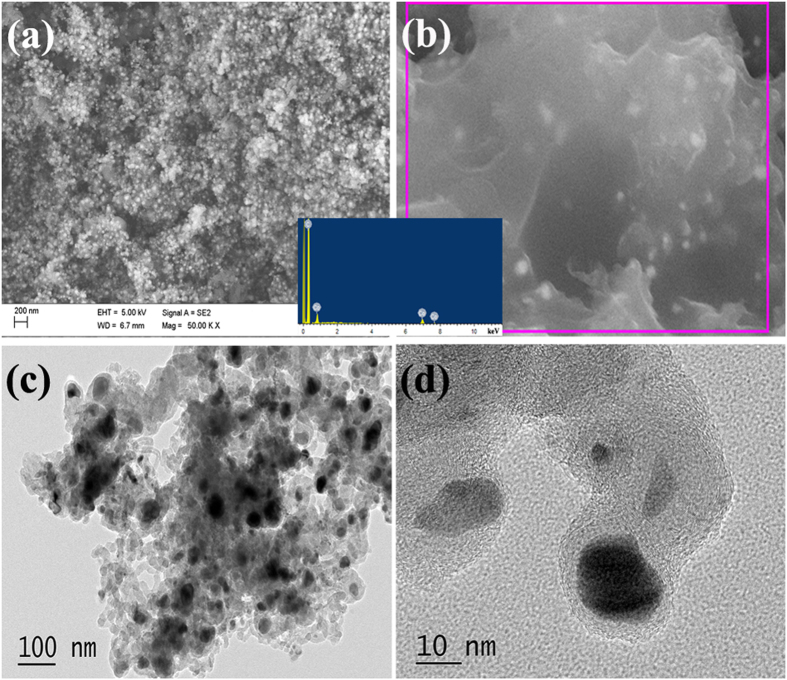
(**a**,**b**) SEM (inset: EDS result), and (**c**,**d**) TEM images of G400.

Figure 3 presents the SEM and TEM images of the obtained G400. As shown in Fig. 3a and b, flexible two-dimensional graphene sheet and CNTs can be found in large scale. Moreover, one can obeserve clearly that Co nanoparticles distribute throughout the obtained G400. As shown in the inset of Fig. 3, the EDS result (obtained from the area as indicated by the red square in Fig. 3b) shows that only C and Co can be detected. The disappearance of O over the as-prepared hybrid is in accordance with the obtained FT-IR results (as shown in Fig. 2d), implying that RGO is reduced to graphene during the CVD process. As shown in Fig. 3c,d, the TEM investigation indicates that Co nanoparticles are encapsulated tightly by CNTs and graphene sheet. Moreover, the AFM images of Co_3_O_4_/RGO and G400 (as shown in Figure S1) also demonstrate that their layered morphology, which is consistent with their TEM and SEM obeservations. Because the thicknesses of RGO and graphene are within a large range (as shown in Figures S1b and d), it is very difficult to determine the variation in thickness during the CVD process. Moreover, similar to that of G400, the SEM and TEM investigations (as shown in Figure S2) shows that two-dimensional graphene sheet and CNTs can also be found in the obtained G450. Compared to that of G400, one can find that much more CNTs exist in the obtained G450. In the experimental section, using the same amount of Co_3_O_4_/RGO as catalyst precursor, larger quantities of G450 could be collected than G400 after cooling to room temperature (RT). Based on the aforementioned results, it can seen that the increase in weights of the collected samples should be ascribed to the growth of CNTs. Therefore, higher content of CNTs can be obtained in G450 than that of G400, which means that CNTs can be observed more easily in G450. Generally, the obtained results indicate that heteronanostructured Co/CNTs-G ternary hybrids can be synthesized in large-scale through the catalytic decomposition of acetylene over Co_3_O_4_/RGO at the designed temperature.

**Figure 4:**
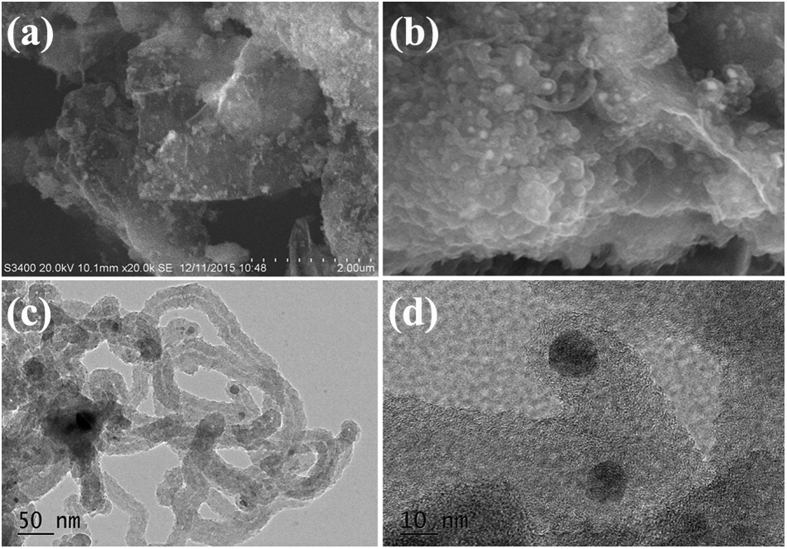
(**a,b**) SEM, and (**c,d**) TEM images of G500.

**Figure 5:**
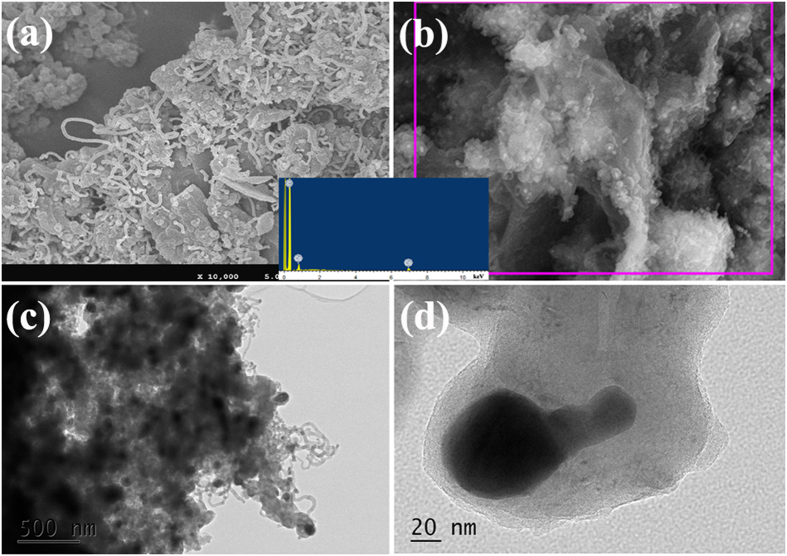
(**a**) SEM (inset: EDS result), and (**b–d**) TEM images of G550.

Figure 4 displays the SEM and TEM images of the as-prepared G500. As shown in Fig. 4a and b, the flexible two-dimensional graphene sheet and CNTs can be observed clearly. The SEM observation indicates that the obtained G450 is also heteronanostructured Co/CNTs-G ternary hybrid. As shown in Fig. 4c, one can observed clearly that the hollow tubular structure of CNTs. Additionally, Co nanoparticles encapsulated at the top of CNTs and inlaid into graphene sheet can also be seen clearly (as shown in Fig. 4c and d). Compared to those of G400 and G450, the SEM and TEM investigations reveal that much higher CNT content can be obtained in G500. Figure 5 presents the microstructure of G550. As shown in Fig. 5a and b, similar to those of G400, G450 and G500, two-dimensional graphene sheet and CNTs can be observed obviously in the obtained sample. As shown in the inset of Fig. 5, the EDS result, which is obtained from the area as indicated by the red square in Fig. 5b, indicates that only C and Co can be detected over G550. Similar to that of G400, the disappearance of O over the as-prepared hybrid further confirms that RGO is reduced further during the CVD process. And the tube structure of CNTs, Co nanoparticles enwrapped by CNTs and graphene sheet can also be seen evidently (as shown in Fig. 5c and d). Differently from those of G400, G450 and G500, the SEM and TEM investigations show that much larger quantities of CNTs can be produced and graphene is seldom seen in the obtained G550. In generally, all the results show that the as-prepared G550 is also heteronanostructured Co/CNTs-G ternary hybrid. Combined with the obtained results, it can be seen clearly that: (1) through the catalytic decomposition of acetylene at 400–550 °C over Co_3_O_4_/RGO, CNTs can be synthesized in large-scale; (2) all the obtained samples are heteronanostructured Co/CNTs-G ternary hybrids; (3) With the rise of temperature, Co/CNTs-G hybrids with much higher CNT content can be obtained. Therefore, a simple and efficient scheme is proposed to synthesize heteronanostructured Co/CNTs-G ternary hybrids.

**Figure 6:**
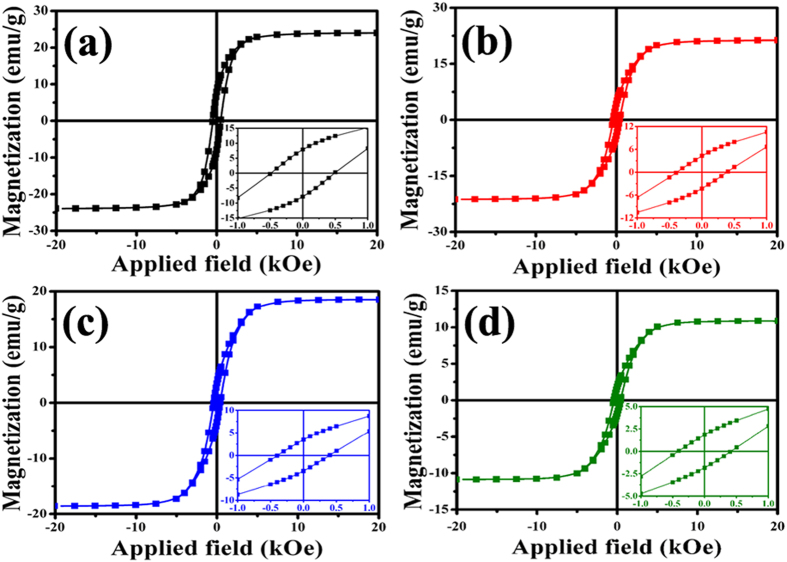
Magnetic hysteresis loop for (**a**) G400, (**b**) G450, (**c**) G500, and (**d**) G550 at RT (inset is the enlarged part close to the origin).

Figure 6 displays the M-H curves of the obtained samples acquired at 300 K. The comparison results reveal that: (1) all the obtained Co/CNTs-G ternary hybrids exhibit typical ferromagnetic properties at RT; (2) as shown in Table 1, one can find that the experiment shows an excellent reproducibility and the saturation magnetization (M_s_) value of the obtained hybrids decreases with the increase of temperature. Based on the aforementioned results, their good ferromagnetic properties at RT should be ascribed to the Co nanoparticles. As revealed in Table 1, over the same amount of Co_3_O_4_/RGO, much larger quantities of CNTs can be synthesized with the increasing temperature. In other words, the Co content in the obtained hybrids decreases with the rise of temperature. Therefore, the obtained M_s_ value of hybrids should be as follows: G400>G450>G500>G550, which is consistent with the obtained M_s_ results (as shown in Table 1). As shown in Fig. 6a and b, the M_s_ values of G400 and G450 are 23.9 and 21.2 emu/g, respectively. Compared to those of magnetic graphene-based hybrids reported before^49^,^50^,^51^, the as-synthesized Co@CNTs-G ternary hybrids display an enhanced magnetic property due to high content of magnetic nanoparticle. Based on the Co content in the obtained hybrids and the M_s_ value of Co at 300 K, the theoretical M_s_ values of G400, G450, G500 and G550 should be 26.1, 14.2, 10.4 and 4.1 emu/g, respectively. The disparity between the calculated and experimental results may be related to nanosize, uneven distribution and different crystallinities of magnetic nanoparticles^52^,^53^. Moreover, it is observed that the obtained Co@CNTs-G ternary hybrids exhibit no changes in XRD patterns and magnetic properties after being kept in air for one month, which confirms further that the ferromagnetic Co nanoparticles are encapsulated tightly in CNTs and graphene sheets. In generally, because of Co nanoparticles tightly wrapped by the graphitic layers, the as-prepared hybrids exhibit good stabilities and magnetic properties at RT, and the M_s_ and H_c_ values can be tuned by the temperature, which may expand their potential application in magnetic date storage and human tumor therapy effectively. Although the introduction of CNTs decreases the M_s_ value of the obtained hybrids, their complex permittivity and chemical stability of Co nanoparticle can be enhanced evidently, which is conducive to obtain a good impedance matching and improve their MA capability.

According to the transmission line theory, the values of reflection loss (RL) and attenuation constant (**α**) were calculated by the following equations^54^,^55^:


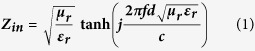



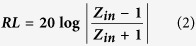



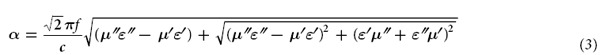


where ***f*** is the frequency of EM wave, ***d*** is the thickness of absorber, ***C*** is the velocity of light and ***Z***_***in***_ is the input impedance of absorber.

**Figure 7:**
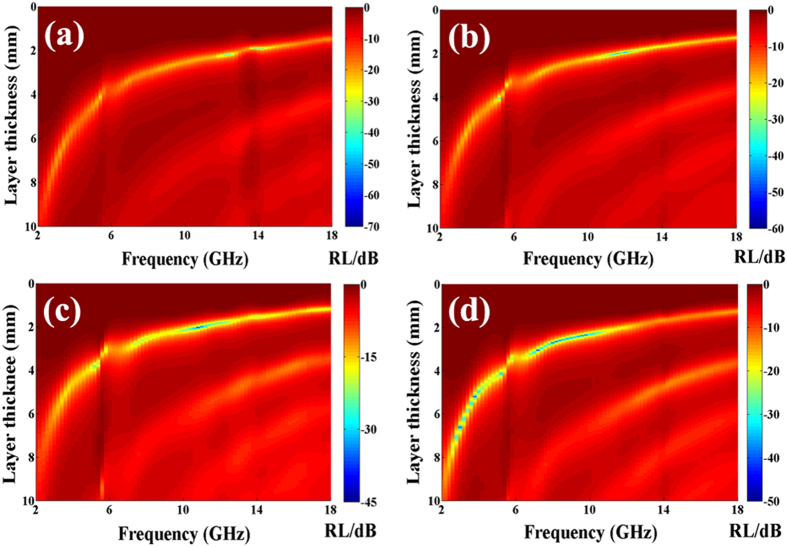
Two-dimensional representation RL values of (**a**) G400, (**b**) G450, (**c**) G500 and (**d**) G550, respectively.

Based on the equations (1) and (2), the RL values of as-synthesized Co/CNTs-G ternary hybrids are obtained and the results are shown in Fig. 7. It can be seen clearly that: (1) the minimum RL value of the obtained hybrids moves towards the lower frequency region with the increasing thickness; (2) the minimum RL values for G400 and G450 are ca −65.6 dB at 12.4 GHz with the matching thickness of 2.19 mm and −58.1 dB at 11.8 GHz with a matching thickness of 1.98 mm, respectively; (3) the optimum RL values for G450 and G500 are ca −41.1 and −47.5 dB at 10.8 and 8.2 GHz with a matching thickness of 2.03 and 2.65 mm, respectively; (4) RL values below −20 dB (99% of EM wave attenuation) for G400, G450, G500 and G550 can be obtained in the frequency range of 2.5–18.0, 5.0–15.8, 5.2–12.6 and 2.6–11.0 GHz, respectively; (5) RL values below −10 dB (90% of EM wave attenuation) for G400, G450, G500 and G550 can be observed in the frequency range of 2.0–18.0, 2.4–18.0, 2.6–18.0 and 2.0–18.0 GHz. Generally, as shown in Table 2, the MA performance of the obtained hybrids is as follows: G400>G450>G550>G500. In addition, due to the synergetic effect of Co nanoparticles, CNTs and graphene, the as-prepared Co/CNTs-G ternary hybrids show the superior absorption properties among other similar hybrids (as illustrated in Table 2).

## Discussion

Based on the aim of experiments and the results obtained, we find that the composition of as-prepared hybrids can be regulated by the pyrolysis temperature. And the formation of heteronanostructured Co@CNTs-G ternary hybrids may be explained by the following reactions:


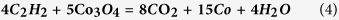



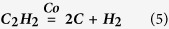


**Figure 8:**
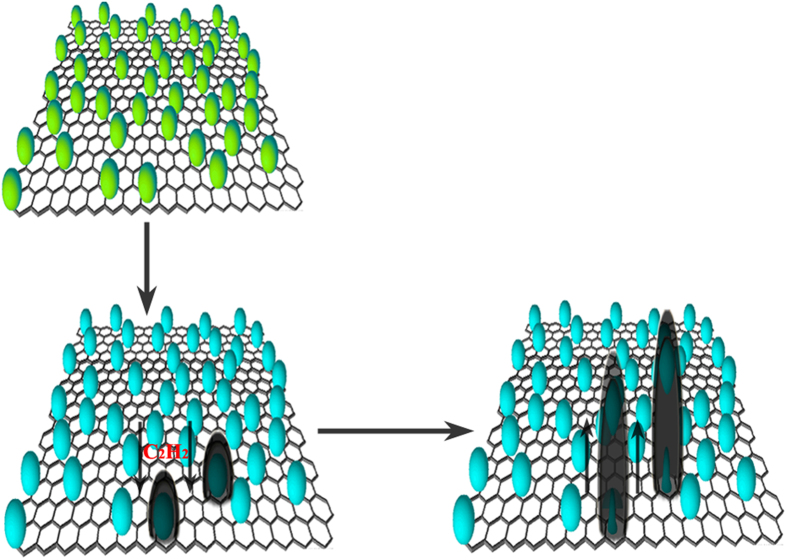
Schematic diagram for the formation mechanism of Co@CNTs-G ternary hybrids.

Among these reactions, reaction (4) is a complete reduction reaction between C_2_H_2_ and Co_3_O_4_, which can occur at the designed temperature (400–550 °C). The elemental of Co can form through the reduction of Co_3_O_4_ by C_2_H_2_. It is well known that the transition metals (such as Fe, Co and Ni) can be used as the effective catalyst for the catalytic decomposition of hydrocarbons (such as methane, acetylene and alcohol) [as shown in reaction (5)] to produce CNTs^66^,^67^,^68^. Therefore, the formation of Co@CNTs-G ternary hybrids should be the result of the reduction reaction between C_2_H_2_ and Co_3_O_4_/RGO. Moreover, according to the obtained results and previously reported models^69^,^70^,^71^, the growth of CNTs over Co_3_O_4_/RGO should follow the tip growth mode and the formation mechanism of Co@CNTs-G ternary hybrids is given in Fig. 8. The possible pathways to grow Co@CNTs-G ternary hybrids are in the following manner: (1) the reduction reaction between Co_3_O_4_ and C_2_H_2_ to form Co nanoparticles; (2) the decomposition of C_2_H_2_ on the Co surface to generate carbon atoms; (3) the dissolution and diffusion of carbon atom around the catalyst; (4) carbon film grows along the surface of catalyst and results in the formation of Co@CNTs-G ternary hybrids.

**Figure 9:**
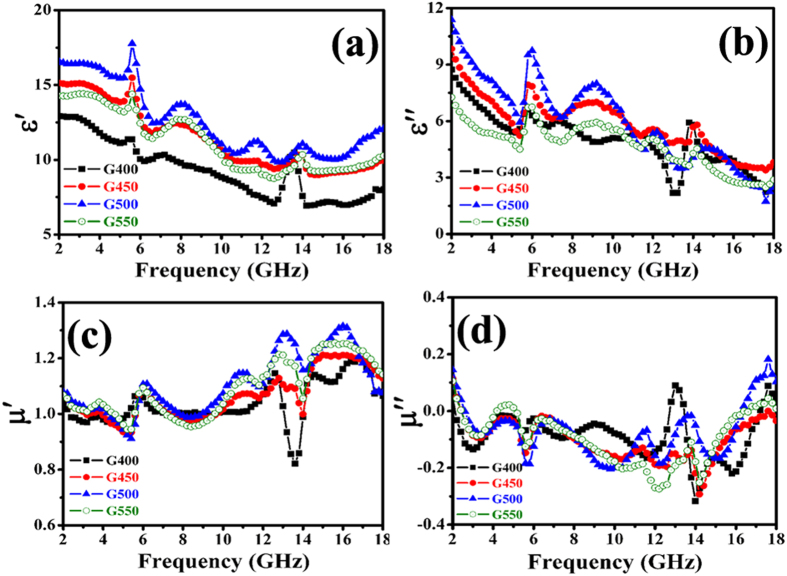
EM parameters of the obtained samples: (**a**) real part, (**b**) imaginary part of permittivity, and (**c**) real part, (**d**) imaginary part of permeability.

In order to analyze the difference in obtained RL results and probable MA mechanism, the EM parameters, dielectric and magnetic loss abilities, attenuation constant and EM impedance matching are presented. Figure 9 gives the complex permittivity and complex permeability of Co/CNTs-G ternary hybrids in the 2.0–18 GHz frequency range. As shown in Fig. 9a and b, the obtained Co/CNTs-G hybrids exhibit the similar trends in the complex permittivity, which confirms further all the obtained samples are the same type of hybrids. Besides some fluctuations, the ***ε***′ and ***ε*****″** values of Co/CNTs-G ternary hybrids are found to decrease with the frequency in the tested region. On the basis of the Debye theory, ***ε***′ and ***ε*****″** can be described as^48^:


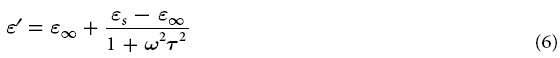



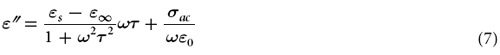


where ***ε***_***s***_ is the static permittivity, ***ε***_***∞***_ is the relative dielectric permittivity at the high frequency limit, ***ω*** is angular frequency, ***τ*** is polarization relaxation time, ***σ***_***ac***_ is the alternative conductivity and ***ε***_***0***_ is the dielectric constant in vacuum. According to the equations (6) and (7), one can find that the decreases of ***ε*****′** and ***ε*****″** are mainly attributed to the increase of ***ω***. As reported previously^42^,^48^, the phenomenon can be considered as the polarization relaxation in the lower frequency range. Similar to the previously reported carbon-based composites^72^, all the obtained hybrids exhibit the resonance peaks at ca. 5.8 and 13.8 GHz, which is favorable to the improvement of MA properties. Moreover, it can be seen that the complex permittivities of the obtained hybrids are as follows: G400<G550<G450<G500, which implies that the increasing CNT content in the obtained hybrid can enhance effectively the values of ***ε***′ and ***ε*****″**, and the relatively high CNT content in the obtained G550 will result in an evident reduction in these values. Similar to GO/CNTs-Fe_3_O_4_ composites^73^, this can be explained that much more CNTs within limits in the obtained hybrids may increase the electric polarization and electric conductivity. Base on the obtained results, one can find that although the M_s_ value of the obtained Co/CNTs-G ternary hybrids decreases with the increase of temperature (as shown in Fig. 6), the introduction of CNTs effectively improves the antioxidant capacity of Co nanoparticles and dielectric loss ability of as-prepared hybrids. Moreover, as we all know that the tube structure is more conducive to absorb EM wave, which is the major purpose of this study to introduce CNTs into the obtained hybrids. Therefore, compared to the previously reported results obtained from Fe@G, Co/G and Ni/G^33^,^62^,^63^, the as-prepared Co/CNTs-G ternary hybrids exhibit enhanced MA capabilities. Figures 9c and d show the complex permeability of Co/CNTs-G ternary hybrids as a function of frequency. Similar to the complex permittivity, the obtained Co/CNTs-G ternary hybrids exhibit the similar trends in the complex permeability. And the ***μ*****″** values of hybrids exhibit a peak at ca. 13.0 GHz, which can be ascribed to the natural resonance as reported elsewhere^11^. Because of the little difference in magnetization, we can find that the discrepancy of complex permeability is unobvious^74^.

Based on the Debye theory, if the second part of equation (7) is not taken into account, the relationship of ***ε*****′** and ***ε*****″** can be written as:


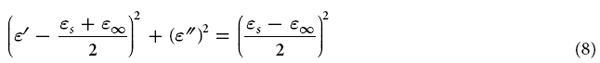


**Figure 10:**
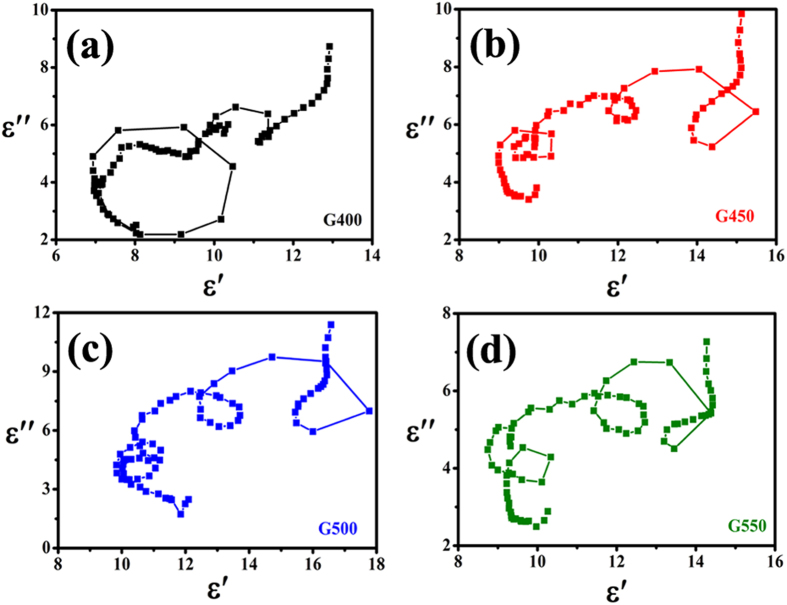
Cole-Cole plots of Co@CNTs-G hybrids: (**a**) G400, (**a**) G450, (**a**) G500 and (**a**) G550.

It corresponds to a circle center at 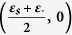, which is characteristic for Debye relaxation process. Therefore, the Debye relaxation process can be reflected in the plot of ***ε*****′** versus ***ε*****″**, and each semicircle corresponds to one Debye relaxation process. As shown in Fig. 10, the Cole-Cole curves of the as-prepared hybrids indicate that each plot of ***ε*****′** versus ***ε*****″** contains many individual semicircles, which means the multirelaxations dielectric properties. According to the previous mechanisms^42^,^75^, the multirelaxations are supported to originate from the multiple interfacial polarizations in the as-prepared Co@CNTs-G ternary hybrids.

**Figure 11:**
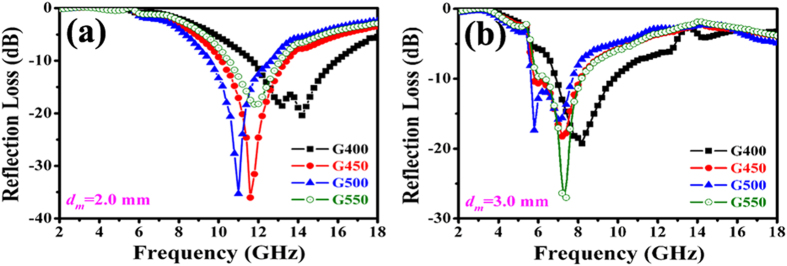
Typical RL values of the obtained samples with the thickness of (**a**) 2.0 mm, and (**b**) 3.0 mm, respectively.

Figure 11 shows a typical RL versus frequency for the as-prepared hybrids with the thickness of 2.0 and 3.0 mm. Besides the obtained G550, it can be seen that the minimum RL value of as-prepared hybrids moves towards the lower frequency region with the increasing temperature. Moreover, as mentioned aforementioned (as shown in Fig. 7), the minimum RL value moves towards the lower frequency region with the increasing thickness. Actually, these phenomena can be explained by the 1/2 wavelength equation:


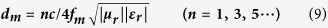


Here, ***d***_***m***_ and ***f***_***m***_ are the matching thickness and frequency of RL peak. In terms of the same sample, one can find that ***d***_***m***_ is inversely proportional to ***f***_***m***_. Therefore, as shown in Fig. 7, the minimum RL value moves towards the lower frequency region with the increasing thickness. For the different samples, when ***d***_***m***_ is kept constant, the complex permittivity and permeability are inversely proportional to ***f***_***m***_. Based on the obtained results (as shown in Fig. 9), one can understand easily that the RL peak moves towards the lower frequency region with the increasing temperature besides the obtained G550. Generally, the obtained results indicate that the scope of absorption can be tuned by regulating CNT content in the obtained hybrids. Moreover, as reported previously^76^,^77^, graphene is more capable of forming conducive paths and the introduction of magnetic nanoparticles can lower the complex permeability of hybrids. Therefore, the addition of graphene and Co nanoparticles in hybrids is very important for the improvement and regulation of their MA properties. Compared the reported data with our obtained results^60^,^61^,^62^, one can find that the enhanced MA performance of as-prepared Co@CNTs-G ternary hybrids is attributed to the compensatory effect of Co nanoparticles, CNTs and graphene.

**Figure 12:**
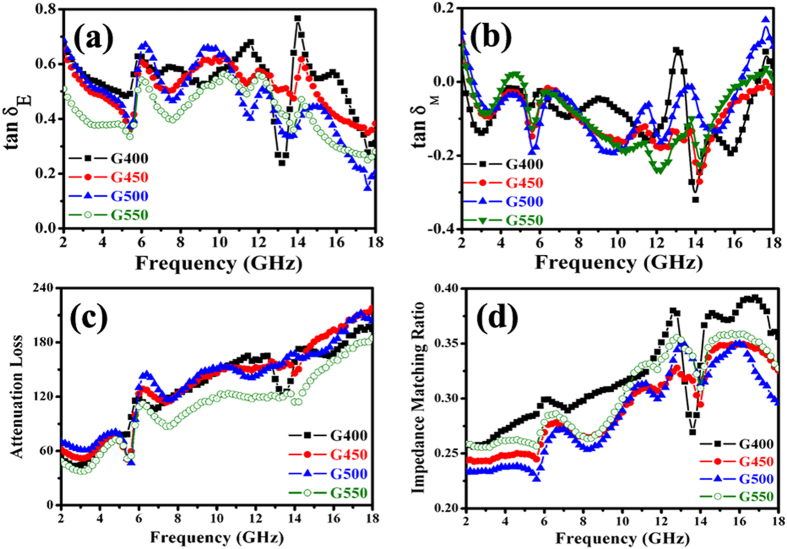
(**a,b**) Loss tangent, (**c**) attenuation loss, and (**d**) impedance matching of the obtained samples.

In order to analyze the possible enhanced MA mechanism, the dielectric and magnetic loss properties, attenuation constant and impedance matching of the obtained nanohybrids were investigated in details. As shown in Fig. 12a and b, one can find that all the obtained hybrids exhibit much larger values of ***tanδ***_***E***_ than those of ***tanδ***_***m***_, which implies that the EM attenuation is mainly due to dielectric loss. And the dielectric loss performance (as shown in Fig. 12a) of the hybrids presents the following tendency: G400>G450>G500>G550. The difference in the values of dielectric loss should be related to the different CNT contents. Moreover, the obtained hybrids display excellent mutual compensation between dielectric loss and magnetic loss, and this effective compensation is very beneficial to enhance their MA capabilities^60^. According to equation (3), the ***α*** values of hybrids were obtained and shown in Fig. 12c. It can be seen that all the as-prepared hybrids exhibit a similar variation curve and the difference in the ***α*** values of G400, G450, G500 and G550 is very small. Actually, the ***α*** value of as-prepared hybrid increases slightly with the rise of CNT content in the hybrid, and the ***α*** value of G550 exhibits an evident fall when the CNT content in the obtained hybrid is high enough. In addition, compared to the previously reported MnO_2_@Fe-G, Fe/MWCNTs, Co/MWCNTs and Ni/MWCNTs^51^,^60^, the ***α*** values of the obtained ternary nanohybrids are much higher, and the high value of ***α*** is conducive to improve EM wave absorption capability^6^. Based on the measured complex permittivity and permeability, the impedance matching ratios of the as-prepared ternary hybrids are obtained and the results are displayed in Fig. 12d. One can find that: (1) similar to the aforementioned results, all the as-prepared hybrids exhibit a similar variation curve; (2) the evident difference in the impedance matching ratios of G400, G450, G500 and G550 can be observed; (3) with the increase of temperature (from 400 to 500 °C), significant decrease can be observed in the impedance matching ratio; (4) the as-prepared G550 reverses the downward trend in the impedance matching ratio. Based on the aforementioned results, one can find that the enhanced MA capabilities of Co/CNTs-G ternary hybrids can be attributed to the good dielectric loss ability, excellent mutual compensation between dielectric loss and magnetic loss, high attenuation constant and good impedance matching. And these good performances are mainly due to the mutual compensation of graphene, CNTs and Co nanoparticles. Based on the previous and obtained results^61^,^62^,^63^, it can seen that the tube and layer structures of CNTs and graphene effectively improve the EM and MA properties of the as-prepared hybrids, and the magnetic Co nanoparticles lower the ***ε***_***r***_ values and improve the equality of ***ε***_***r***_ and ***μ***_***r***_, which helps to enhance the level of impedance matching. The heteronanostructure makes as-prepared Co/CNTs-G ternary hybrids show an excellent mutual compensation between dielectric loss and magnetic loss.

Recently, two models have been proposed to interpret enhanced EM wave absorption properties of hybrids^6^,^78^. The first one is geometrical effect, which occurs when the incident and reflected waves in the material are out of phase 180° at the particular thickness. This effect is strongly dependent on the aforementioned equation (9). According to the obtained values of RL, ***f***_***m***_, ***μ***_***r***_ and ***ε***_***r***_, the matching thickness (***d***_***m***_) can be obtained. As shown in Table 3, we can find that the calculated matching thickness ***d***_***m***_ is not absolutely equal to the true thickness ***d***. Therefore, the geometric effect cannot account for their excellent EM wave absorption properties very well. The other model is zero reflection, according to the EM wave theory, the relationship **μ**_**r**_ = ***ε***_***r***_ should be satisfied. However, as shown in Fig. 9, all the obtained samples exhibit much higher values of permittivity than their permeability. Therefore, the model cannot be used to explain the obtained results. Based on the aforementioned results, the excellent MA properties of Co@CNTs-G ternary hybrids should be related to their special structure and synergetic effect. First, the as-prepared Co@CNTs-G hybrids simultaneously possess the tube and two dimensional layer structures (as shown in Figs 3, 4, 5). It is well known that these structures are favorable to absorb the incident EM wave. Second, due to their heteronanostructure, the multiple interfacial polarizations exist in the as-prepared Co@CNTs-G hybrids (as shown in Fig. 10), which contributes to the improved EM wave absorbing capability^51^,^79^. Third, the obtained ternary hybrids consist of magnetic (Co nanoparticles) and dielectric loss (CNTs and graphene) materials. The heteronanostructure makes the hybrids exhibit high attenuation constant, good impedance matching and excellent mutual compensation between dielectric loss and magnetic loss (as shown in Fig. 12), which are helpful for the improvement of EM wave absorption properties.

In summary, in order to explore high efficiency MAMs, we designed and synthesized heteronanostructured Co@CNTs-G ternary hybrids through catalytic decomposition of acetylene at the designed temperature over Co_3_O_4_/RGO. The CNT content in the as-prepared ternary hybrids could be regulated by controlling the temperature. The investigations indicated that the heteronanostructured Co@CNTs-G ternary hybrids exhibited excellent MA properties, and their EM and MA properties could be tuned by the CNT content. Moreover, the obtained results showed that the enhanced MA performance of the as-prepared Co@CNTs-G ternary hybrids could be ascribed to their special structure and synergetic effect, which made the obtained ternary hybrids exhibit good dielectric loss ability, excellent mutual compensation between dielectric loss and magnetic loss, high attenuation constant and good impedance matching.

## Methods

### Material preparation

Co_3_O_4_/reduced graphene oxide (Co_3_O_4_/RGO) was purchased from XFNANO Materials Tech Co., Nanjing, China. The chemical regents were analytically pure and used without further purification. Initially, 0.02 g of Co_3_O_4_/RGO was dispersed on a ceramic plate which was placed inside a quartz reaction tube and the tube furnace was heated from RT to the designed temperature (400, 450, 500 and 550 °C) in argon, respectively. Subsequently, acetylene was introduced into the reaction tube furnace at atmospheric pressure, and the pyrolysis of acetylene was carried out at a selected temperature for 2 h. Finally, after cooling to RT in argon, about 0.033, 0.055, 0.075 and 0.1893 g of products (black in color) were collected on the plate at 400, 450, 500 and 550 °C, respectively. For easy description, the samples obtained at 400, 450, 500 and 550 °C were denoted as G400, G450, G500 and G550, respectively.

### Measurement

The samples were examined on an X-ray powder diffractometer (XRD) at RT for phase identification using CuK_α_ radiation (model D/Max-RA, Rigaku). Raman spectroscopic investigation was performed using a Jobin-Yvon Labram HR800 instrument with 514.5 nm Ar^+^ laser excitation. The morphology investigations were examined using a transmission electron microscope (TEM) (model Tecnai-G20, operated at an accelerating voltage of 20 kV), and field emission scanning electron microscope (FE-SEM) (model ZEISS SUPRA-40, JSM-7500F and S3400, operated at accelerating voltages of 5 kV). Fourier transform infrared (FT-IR) spectroscopy of samples (in KBr pellets) was recorded using a Nicolet 510 P spectrometer. Atomic force microscopy (AFM) measurements were carried out by using Nanonics MV4000. The magnetic properties of samples were measured at 300 K using a Quantum Design MPMS SQUID magnetometer (Quantum Design MPMS-XL) equipped with a superconducting magnet capable of producing fields of up to 50 kOe. For microwave measurement, 30 wt% of the as-prepared sample was mixed with paraffin and pressed into coaxial clapper in a dimension of outer diameter of 7.0 mm, inner diameter of 3.0 mm, respectively. The complex permittivity 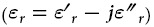 and complex permeability 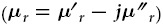 of the composite were measured in frequency range of 2–18 GHz over an Agilent E8363B vector network analyzer.

## Additional Information

**How to cite this article**: Qi, X. *et al*. Heteronanostructured Co@carbon nanotubes-graphene ternary hybrids: synthesis, electromagnetic and excellent microwave absorption properties. *Sci. Rep.* **6**, 37972; doi: 10.1038/srep37972 (2016).

**Publisher's note:** Springer Nature remains neutral with regard to jurisdictional claims in published maps and institutional affiliations.

## Supplementary Material

Supplementary Information

## Figures and Tables

**Table 1:** Effects of pyrolysis temperature on products.

Temperature (°C)	Product	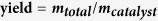	M_s_ value (emu/g)
400	G400	1.7	23.9
		1.5	
		1.8	
550	G450	2.8	21.2
		2.8	
		2.7	
500	G500	3.7	18.6
		3.8	
		3.8	
550	G550	9.6	10.9
		9.3	
		9.5	

**Table 2:** EM wave absorption properties of carbon-based and graphene-based hybrids reported in recent representative papers.

MAMs	Optimum RL (dB)	Optimum thickness (mm)	Frequency range (GHz) (RL<−20 dB)	Reference
Co@C	−52	3	4.0–18.0	56
Ni/C	−45	2	4.0–14.2	57
(Fe, Ni)/C	−26.9	2	13.6–16.6	58
Fe/CNTs	−25	1.2	7.0–15.2	59
Fe/MWCNTs^a^	−39	4.27	2.04–3.47	60
Co/MWCNTs	−37	5.25	2.35–3.51	60
Ni/MWCNTs	−37	5.19	1.83–3.07	60
G/CNTs	−44.6	3	6.0–9.0	61
Fe@G	−45	3.0	4.0–18.0	33
Co/G	−47.5	2.0	3.0–13.0	62
Ni/G	−17.8	5.0	–	63
NiFe_2_O_4_@ G	−29.2	2.0	5.7–17.5	35
MnO_2_@Fe-G	−17.5	1.5	–	51
ZnO/Fe@Fe_3_O_4_/G	−38.4	5.0	5.9–15.2	64
G@Fe_3_O_4_@ SiO_2_@NiO	−51.5	1.8	12.0–15.0	65
G400	−65.6	2.19	2.5–18.0	this work
G450	−58.1	1.98	5.0–15.8	this work
G500	−41.1	2.03	5.2–12.6	this work
G550	−47.5	2.65	2.6–11.0	this work

^a^Fe/multiwalled carbon nanotubes.

**Table 3:** RL values of the obtained nanohybrids at the calculated matching thickness (***d***_***m***_) and the real thickness (***d***) in terms of geometrical effect.

Sample	*d*/mm	RL/dB	*f*_*m*_/GHz	*d*_*m*_/mm
G400	1.5	−18.9	17.8	1.4
	2.0	−20.3	14.2	1.7
	2.5	−21.0	10.2	2.3
G450	1.5	−20.1	15.4	1.4
	2.0	−36.1	11.6	1.9
	2.5	−18.6	8.6	2.4
G500	1.5	−18.4	14.0	1.4
	2.0	−35.3	11.0	1.8
	2.5	−19.3	8.0	2.4
G550	1.5	−13.3	15.4	1.4
	2.0	−18.5	11.8	1.8
	2.5	−32.1	8.8	2.4

## References

[b1] Girgert R., Grundker C., Emons G. & Hanf V. Electromagnetic fields alter the expression of estrogen receptor cofactors in breast cancer cells. Bioelectromagnetics 29, 169–176 (2008). 18027843 10.1002/bem.20387

[b2] Liu X. G. . Dual nonlinear dielectric resonance and strong natural resonance in Ni/ZnO nanocapsules. Appl. Phys. Lett. 94, 053119 (2009).

[b3] Wang Y. M., Li T. X., Zhao L. F., Hu Z. W. & Gu Y. J. Research progress on nanostructured radar absorbing materials. Energy Power Eng. 3, 580–584 (2011).

[b4] Qin F. & Peng H. X. Ferromagnetic microwires enabled multifunctional composite materials. Prog. Mater. Sci. 58, 183–259 (2013).

[b5] Michielssen E., Sager J. M., Ranjithan S. & Mittra R. Design of lightweight, broad-band microwave absorbers using genetic algorithms. IEEE Trans. Microw. Theory Tech. 41, 1024–1031 (1993).

[b6] Yusoff A. N. . Electromagnetic and absorption properties of some microwave absorbers. J. Appl. Phys. 92, 876–882 (2002).

[b7] Cui C. K. . Synthesis of electromagnetic functionalized Fe_3_O_4_ microspheres/polyaniline composites by two-step oxidative polymerization. J. Phys. Chem. B 116, 9523–9531 (2012). 22800337 10.1021/jp3024099

[b8] Liu Q. H. . Insights into size-dominant magnetic microwave absorption properties of CoNi microflowers via off-axis electron holography. ACS Appl. Mater. Inter. 7, 4233–4240 (2015). 10.1021/am508527s 25642817

[b9] Saini P. . High permittivity polyaniline-barium titanate nanocomposites with excellent electromagnetic interference shielding response. Nanoscale 5, 4330–4336 (2013). 23563991 10.1039/c3nr00634d

[b10] Sun G., Dong B., Cao M., Wei B. & Hu C. Hierarchical dendrite-like magnetic materials of Fe_3_O_4_, γ-Fe_2_O_3_, and Fe with high performance of microwave absorption. Chem. Mater. 23, 1587–1593 (2011).

[b11] Liu X. G., Geng D. Y., Meng H., Shang P. J. & Zhang Z. D. Microwave-absorption properties of ZnO-coated iron nanocapsules. Appl. Phys. Lett. 92, 173117 (2008).

[b12] Ren Y. L. . Three-dimensional SiO_2_@Fe_3_O_4_ core/shell nanorod array/graphene architecture: synthesis and electromagnetic absorption properties. Nanoscale 5, 12296–12303 (2013). 24154630 10.1039/c3nr04058e

[b13] Liu J. W. . Double-shelled yolk–shell microspheres with Fe_3_O_4_ cores and SnO_2_ double shells as high-performance microwave absorbers. J. Phys. Chem. C 117, 489–495 (2013).

[b14] Lv H. L. . FeCo/ZnO composites with enhancing microwave absorbing properties: effect of hydrothermal temperature and time. RSC Adv. 4, 57529–57533 (2014).

[b15] Zhao B., Shao G., Fan B., Zhao W. & Zhang R. Investigation of the electromagnetic absorption properties of Ni@TiO_2_ and Ni@SiO_2_ composite microspheres with core-shell structure. Phys. Chem. Chem. Phys. 17, 2531–2539 (2015). 25494450 10.1039/c4cp05031b

[b16] Jian X. . Facile synthesis of Fe_3_O_4_/GCs composites and their enhanced microwave absorption properties. ACS Appl. Mater. Inter. 8, 6101–6109 (2016). 10.1021/acsami.6b00388 26890224

[b17] Saini P., Choudhary V., Vijayan N. & Kotnala R. K. Improved electromagnetic interference shielding response of poly(aniline)-coated fabrics containing dielectric and magnetic nanoparticles. J. Phys. Chem. C 116, 13403–13412 (2012).

[b18] Lv H. L. . Co_x_Fe_y_@C composites with tunable atomic ratios for excellent electromagnetic absorption properties. Sci. Rep. 5, 18249 (2015). 26659124 10.1038/srep18249 PMC4676003

[b19] Jani R. K. & Kumar S. R. N. Microwave absorbing properties of a thermally reduced graphene oxide/nitrile butadiene rubber composite original. Carbon 50, 2202–2208 (2012).

[b20] Novoselov K. S. . Room-temperature electric field effect and carrier-type inversion in graphene films. Science 306, 666–669 (2004). 15499015 10.1126/science.1102896

[b21] Dikin D. A. . Preparation and characterization of graphene oxide paper. Nature 448, 457–460 (2007). 17653188 10.1038/nature06016

[b22] Pei S. & Cheng H. M. The reduction of graphene oxide. Carbon 50, 3210–3228 (2012).

[b23] Kuila T. . Chemical functionalization of graphene and its applications. Prog. Mater. Sci. 57, 1061–1105 (2012).

[b24] Zhou L. . Stable Cu_2_O nanocrystals grown on functionalized graphene sheets and room temperature H_2_S gas sensing with ultrahigh sensitivity. Nanoscale 5, 1564–1569 (2013). 23325161 10.1039/c2nr33164k

[b25] Georgiou T. . Vertical field-effect transistor based on graphene-WS_2_ heterostructures for flexible and transparent electronics. Nat. Nanotechnol. 8, 100–103 (2013). 23263726 10.1038/nnano.2012.224

[b26] Chen S., Zhu J., Wu X., Han Q. & Wang X. Graphene oxide-MnO_2_ nanocomposites for supercapacitors. ACS Nano 4, 2822–2830 (2010). 20384318 10.1021/nn901311t

[b27] Wang C. . The electromagnetic property of chemically reduced graphene oxide and its application as microwave absorbing material. Appl. Phys. Lett. 98, 072906 (2011).

[b28] Hu C. . 3D graphene-Fe_3_O_4_ nanocomposites with high-performance microwave absorption. Phys. Chem. Chem. Phys. 15, 13038–13043 (2013). 23817632 10.1039/c3cp51253c

[b29] Kong L. . Electromagnetic wave absorption properties of reduced graphene oxide modified by maghemite colloidal nanoparticle clusters. J. Phys. Chem. C 117, 19701–19711 (2013).

[b30] Giovannetti G. . Doping graphene with metal contacts. Phys. Rev. Lett. 101, 026803 (2008). 18764212 10.1103/PhysRevLett.101.026803

[b31] Khomyakov P. A. . First-principles study of the interaction and charge transfer between graphene and metals. Phys. Rev. B 79, 195425 (2009).

[b32] Zhang X. J. . Enhanced microwave absorption property of reduced graphene oxide (RGO)-MnFe_2_O_4_ nanocomposites and polyvinylidene fluoride. ACS Appl. Mater. Inter. 6, 7471–7478 (2014). 10.1021/am500862g 24779487

[b33] Zhao X. C. . Excellent microwave absorption property of graphene-coated Fe nanocomposites. Sci. Rep. 3, 3421 (2013). 24305606 10.1038/srep03421 PMC3852363

[b34] Qu B., Zhu C. L., Li C. Y., Zhang X. T. & Chen Y. J. Coupling hollow Fe_3_O_4_-Fe nanoparticles with graphene sheets for high-performance electromagnetic wave absorbing material. ACS Appl. Mater. Inter. 8, 3730–3735 (2016). 10.1021/acsami.5b12789 26829291

[b35] Fu M., Jiao Q. Z. & Zhao Y. Preparation of NiFe_2_O_4_ nanorodgraphene composites via an ionic liquid assisted one-step hydrothermal approach and their microwave absorbing properties. J. Mater. Chem. A 1, 5577–5586 (2013).

[b36] Liu P. & Huang Y. Synthesis of reduced graphene oxide-conducting polymers-Co_3_O_4_ composites and their excellent microwave absorption properties. RSC Adv. 3, 19033–19039 (2013).

[b37] Wang L. . Synthesis and microwave absorption enhancement of graphene@Fe_3_O_4_@SiO_2_@NiO nanosheet hierarchical structures. Small 6, 3157–3164 (2014). 10.1039/c3nr05313j 24496379

[b38] Ritter U. . Radiation damage to multi-walled carbon nanotubes and their raman vibrational modes. Carbon 44, 2694–2700 (2006).

[b39] Thomsen C. & Reich S. Double resonant Raman scattering in graphite. Phys. Rev. Lett. 85, 5214–5217 (2000). 11102224 10.1103/PhysRevLett.85.5214

[b40] Tuinstra F. & Koenig J. L. Raman spectrum of graphite. J. Chem. Phys. 53, 1126–1130 (1970).

[b41] Ferrari A. C. Raman spectroscopy of graphene and graphite: disorder, electro-phonon coupling, doping and nonadiabatic effects. Solid State Commun. 143, 47–57 (2007).

[b42] Wang Y. F. . Hybrid of MoS_2_ and reduced graphene oxide: a lightweight and broadband electromagnetic wave absorber. Appl. Mater. Inter. 7, 26226–26234 (2015). 10.1021/acsami.5b08410 26575796

[b43] Qi X. S., Zhong W., Deng Y., Au C. T. & Du Y. W. Characterization and magnetic properties of helical carbon nanotubes and carbon nanobelts synthesized in acetylene decomposition over Fe-Cu nanoparticles at 450 °C. J. Phys. Chem. C 113, 15934–15940 (2009).

[b44] Qi X. S., Zhong W., Deng Y., Au C. T. & Du Y. W. Synthesis of helical carbon nanotubes, worm-like carbon nanotubes and nanocoils at 450 °C and their magnetic properties. Carbon 48, 365–376 (2010).

[b45] Qi X. S., Ding Q., Zhong W., Au C. T. & Du Y. W. Controllable synthesis and purification of carbon nanofibers and nanocoils over water-soluble NaNO_3_. Carbon 56, 383–385 (2013). 10.1186/1556-276X-8-545 PMC3877971 24369821

[b46] Xue L. P. . Hydrothermal synthesis of graphene-ZnS quantum dot nanocomposites. Mater. Lett. 65, 198–200 (2011).

[b47] Singh K. V. . Microwave absorbing properties of a thermally reduced graphene oxide/nitrile butadiene rubber composite. Carbon 50, 2202–2208 (2012).

[b48] Wu F., Xie A. M., Sun M. X., Wang Y. & Wang M. Y. Reduced graphene oxide (RGO) modified spongelike polypyrrole (PPy) aerogel for excellent electromagnetic absorption. J. Mater. Chem. A 3, 14358–14369 (2015).

[b49] Wang T. S. . Graphene-Fe_3_O_4_ nanohybrids: synthesis and excellent electromagnetic absorption properties. J. Appl. Phys. 113, 024314 (2013).

[b50] Zhang X., Huang Y., Chen X. F., Li C. & Chen J. J. Hierarchical structures of graphene@CoFe_2_O_4_@SiO_2_@TiO_2_ nanosheets: synthesis and excellent microwave absorption properties. Mater. Lett. 158, 380–383 (2015).

[b51] Lv H. L., Ji G. B., Liang X. H., Zhang H. Q. & Du Y. W. A novel rod-like MnO_2_@Fe loading on graphene giving excellent electromagnetic absorption properties. J. Mater. Chem. C. 3, 5056–5064 (2015).

[b52] Ramazani A., Almasi Kashi M., Bayzi Isfahani V. & Ghaffari M. The influence of crystallinity enhancement on the magnetic properties of ac electrodeposited Fe nanowires. Appl. Phys. A 98, 691–697 (2010).

[b53] Singh G. . Tunability in crystallinity and magnetic properties of core-shell Fe nanoparticles. Part. Part. Syst. Char. 31, 1054–1059 (2014).

[b54] Michielssen E., Sager J. M., Ranjithan S. & Mittra R. Design of lightweight, broad-band microwave absorbers using genetic algorithms. Microwave Theory Tech. 41, 1024–1031 (1993).

[b55] Yusoff A. N. . Electromagnetic and absorption properties of some microwave absorbers. J. Appl. Phys. 92, 876–882 (2002).

[b56] Zhang D. F., Xu F. X., Lin J., Yang Z. D. & Zhang M. Electromagnetic characteristics and microwave absorption properties of carbon-encapsulated cobalt nanoparticles in 2-18-GHz frequency range. Carbon 80, 103–111 (2014).

[b57] Zhao H. B., Fu Z. B., Chen H. B., Zhong M. L. & Wang C. Y. Excellent electromagnetic absorption capability of Ni/carbon based conductive and magnetic foams synthesized via a green one pot route. ACS Appl. Mater. Inter. 8, 1468–1477 (2016). 10.1021/acsami.5b10805 26710881

[b58] Liu X. G. . (Fe, Ni)/C nanocapsules for electromagnetic-wave-absorber in the whole Ku-band. Carbon 47, 470–474 (2009).

[b59] Che R. C., Peng L. M., Duan X. F., Chen Q. & Liang X. L. Microwave absorption enhancement and complex permittivity and permeability of Fe encapsulated within carbon nanotubes. Adv. Mater. 16, 401–405 (2004).

[b60] Wen F. S., Zhang F. & Liu Z. Y. Investigation on microwave absorption properties for multiwalled carbon nanotubes/Fe/Co/Ni nanopowders as lightweight absorbers. J. Phys. Chem. C 115, 14025–14030 (2011).

[b61] Wang L., Huang Y., Li C., Chen J. J. & Liu Z. Y. A facile one-pot method to synthesize a three-dimensional graphene@carbon nanotube composite as a high-efficiency microwave absorber. Phys. Chem. Chem. Phys. 17, 2228–2234 (2015). 25485522 10.1039/c4cp04745a

[b62] Pan G. H., Zhu J., Ma S. L., Sun G. B. & Yang X. J. Enhancing the electromagnetic performance of Co through the phase-controlled synthesis of hexagonal and cubic Co nanocrystals grow on graphene. ACS Appl. Mater. Inter. 5, 12716–12724 (2013). 10.1021/am404117v 24266516

[b63] Chen T. T. . Hexagonal and cubic Ni nanocrystals grown on graphene: phase-controlled synthesis, characterization and their enhanced microwave absorption properties. J. Mater. Chem. 22, 15190–15197 (2012).

[b64] Ren Y. L. . Quaternary nanocomposites consisting of graphene, Fe_3_O_4_@Fe core@shell, and ZnO nanoparticles: synthesis and excellent electromagnetic absorption properties. ACS Appl. Mater. Inter. 4, 6436–6442 (2012). 10.1021/am3021697 23176086

[b65] Wang L. . Synthesis and microwave absorption enhancement of graphene@Fe_3_O_4_@SiO_2_@NiO nanosheet hierarchical structures. Nanoscale 6, 3157–3164 (2014). 24496379 10.1039/c3nr05313j

[b66] Andrew R., Jacques D., Qian D. L. & Rantell T. Multiwall carbon nanotubes: synthesis and application. Acc. Chem. Res. 35, 1008–1017 (2002). 12484788 10.1021/ar010151m

[b67] Banks C. E., Crossley A., Salter C., Wilkins S. J. & Compton R. G. Carbon nanotubes contain metal impurities which are responsible for the “electrocatalysis” seen at some nanotube modified electrodes. Angew. Chem. Int. Ed. 45, 2533–2537 (2006). 10.1002/anie.200600033 16544355

[b68] Esconjauregui S., Whelan C. M. & Maex K. The reasons why metals catalyze the nucleation and growth of carbon nanotubes and other carbon nanomorphologies. Carbon 47, 659–669 (2009).

[b69] Amelickx S. . A formation mechanism for catalytically grown helix-shaped graphite nanotubes. Science 265, 635–639 (1994). 17752760 10.1126/science.265.5172.635

[b70] Fan S. . Self-oriented regular arrays of carbon nanotubes and their field emission properties. Science 283, 512–514 (1999). 9915692 10.1126/science.283.5401.512

[b71] Gamaly E. G. & Ebbesen T. W. Mechanism of carbon nanotubes formation in the arc-discharge. Phys. Rev. B 52, 2083–2089 (1995). 10.1103/physrevb.52.2083 9981282

[b72] Sun Y. P. . A facile route to carbon-coated vanadium carbide nanocapsules as microwave absorbers. RSC Adv. 3, 18082–18086 (2013).

[b73] Wang L. N. . Synthesis and microwave absorption property of flexible magnetic film based on graphene oxide/carbon nanotubes and Fe_3_O_4_ nanoparticles. J. Mater. Chem. A 2, 14940–14946 (2014).

[b74] Yang Z. H., Li Z. W., Yang Y. H. & Xu Z. C. J. Optimization of Zn_x_Fe_3-x_O_4_ hollow spheres for enhanced microwave attenuation. ACS Appl. Mater. Inter. 6, 21911–21915 (2014). 10.1021/am5075612 25496607

[b75] Yang H. . NiO hierarchical nanorings on SiC: Enhancing relaxation to tune microwave absorption at elevated temperature. ACS Appl. Mater. Inter. 7, 7073–7077 (2015). 10.1021/acsami.5b01122 25806666

[b76] Khurram A. A., Rakha A., Zhou S., , Shafi M. & Munir A. Correlation of electrical conductivity, dielectric properties, microwave absorption, and matrix properties of composites filled with graphene nanoplatelets and carbon nanotubes. J. Appl. Phys. 118, 044105 (2015).

[b77] Zong M., Huang Y., Zhang N. & Wu H. W. Influence of (RGO)/(ferrite) ratios and graphene reduction degree on microwave absorption properties of graphene composites. J. Alloys Comp. 644, 491–501 (2015).

[b78] Liu J. W. . Microwave absorption enhancement of multifunctional composite microspheres with spinel Fe_3_O_4_ cores and anatase TiO_2_ shells. Small 8, 1214–1221 (2012). 22331748 10.1002/smll.201102245

[b79] Yu H. L. . Graphene/polyaniline nanorod arrays: synthesis and excellent electromagnetic absorption properties. J. Mater. Chem. 22, 21679–21685 (2012).

