# Supplementary material for: Heteronanostructured Co@carbon nanotubes-graphene ternary hybrids: synthesis, electromagnetic and excellent microwave absorption properties
Source: Sci Rep. 2016 Nov 28;6:37972. doi: 10.1038/srep37972 (PMC5125104; doi:10.1038/srep37972)
Supplement: Supplementary Information [file srep37972-s1.doc]

**Electronic Supplementary Information**

**Heteronanostructured Co@carbon nanotubes-graphene ternary hybrids: synthesis, electromagnetic and excellent microwave absorption properties**

Xiaosi Qi^a,b,*^, Qi Hu^a^, Hongbo Cai^a^, Ren Xie^a^, Zhongchen Bai^a^, Yang Jiang^a^, Shuijie Qin^a^, Wei Zhong^b,*^, Youwei Du^b^

*^a^College of Physics, Guizhou University, Guiyang 550025, People’s Republic of China*

*^b^Collaborative Innovation Center of Advanced Microstructures, Nanjing National Laboratory of Microstructures and Jiangsu Provincial Laboratory for NanoTechnology, Nanjing University, Nanjing 210093, People’s Republic of China*


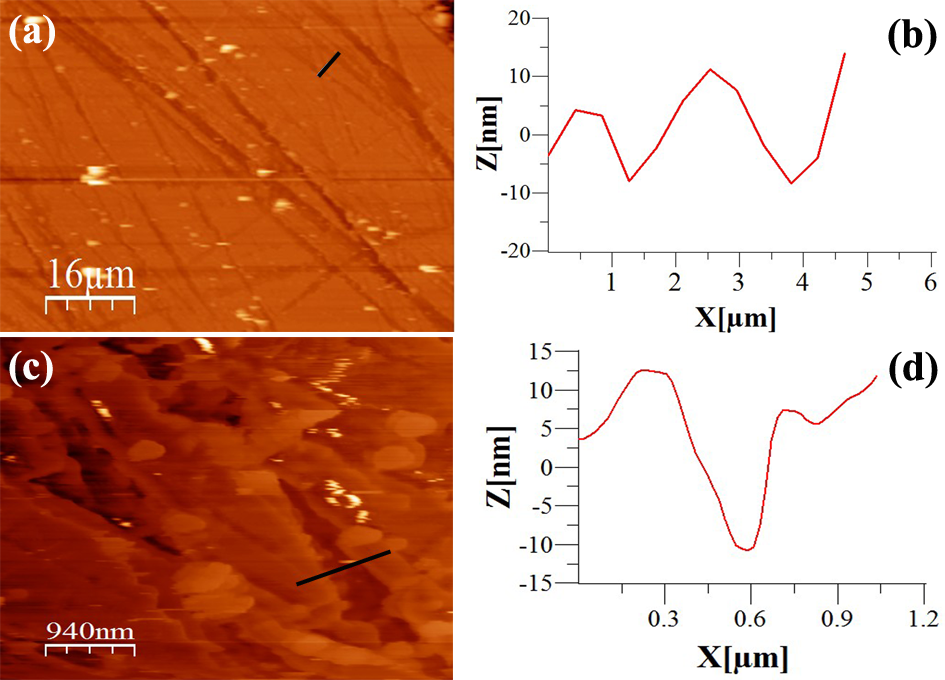


**Figure S1.** AFM images and high profiles of (a, b) Co_3_O_4_/RGO, and (c,d) G400.


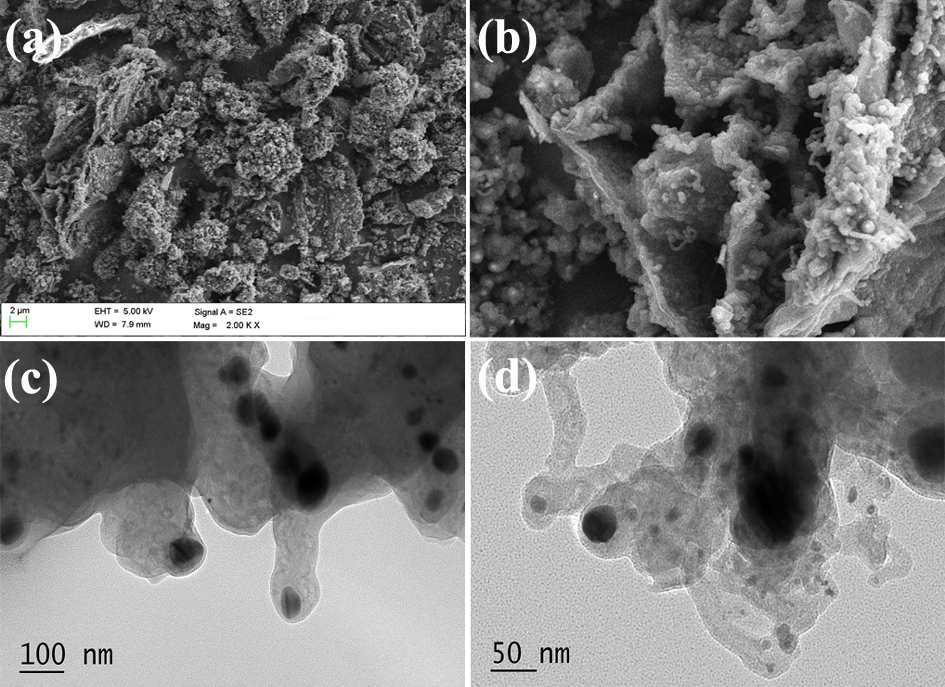


**Figure S2.** (a,b) SEM, and (c,d) TEM images of G450.
